# Draft genome sequence of *Brevibacterium epidermidis* EZ-K02 isolated from nitrocellulose-contaminated wastewater environments

**DOI:** 10.1016/j.dib.2017.12.053

**Published:** 2018-01-03

**Authors:** Elvira E. Ziganshina, Waleed S. Mohammed, Swapnil P. Doijad, Elena I. Shagimardanova, Natalia E. Gogoleva, Ayrat M. Ziganshin

**Affiliations:** a Department of Microbiology, Institute of Fundamental Medicine and Biology, Kazan (Volga Region) Federal University, Kremlyovskaya str. 18, Kazan 420008, Russia; b Department of Biotechnology, Faculty of Agriculture, Al-Azhar University, Cairo 11651, Egypt; c Institute of Medical Microbiology, ​Justus-Liebig University, Giessen 35392, Germany; d Laboratory of Extreme Biology, Institute of Fundamental Medicine and Biology, Kazan (Volga Region) Federal University, Kazan 420021, Russia

**Keywords:** *Brevibacterium epidermidis*, Draft genome, Wastewater

## Abstract

*Brevibacterium* spp. are aerobic, nonbranched, asporogenous, gram-positive, rod-shaped bacteria which may exhibit a rod-coccus cycle when cells get older and can be found in various environments. ​Several *Brevibacterium* species have industrial importance and are capable of biotransformation of various contaminants. Here we describe the draft genome sequence of *Brevibacterium epidermidis* EZ-K02 isolated from nitrocellulose-contaminated wastewater environments. The genome comprises 3,885,924 bp, with a G + C content of 64.2%. This whole genome shotgun project has been deposited at DDBJ/ENA/GenBank under the accession PDHL00000000.

**Specifications Table**

**Table:** 

Subject area	Biology
More specific subject area	Genome analysis
Type of data	Table, figures
How data was acquired	Illumina Miseq
Data format	Analyzed
Experimental factors	Genomic DNA from pure culture
Experimental features	Isolation of bacteria, genome sequencing, draft genome assembly and annotation
Data source location	Nitrocellulose-contaminated wastewater environments, Kazan, Russia
Data accessibility	Data are in public repository. This whole genome shotgun project has been deposited at DDBJ/ENA/GenBank under the accession PDHL00000000 (https://www.ncbi.nlm.nih.gov/nuccore/PDHL00000000). The 16S rRNA gene sequence has been deposited at GenBank under the accession number MG050737 (https://www.ncbi.nlm.nih.gov/nuccore/MG050737).

**Value of the data**

• Several *Brevibacterium* species have industrial importance and are capable of biotransformation of various contaminants; therefore, more investigations at the genomic level are necessary to improve our understanding of their ecology, genetics, as well as potential biotechnological applications.
• Data shown here can be useful for other groups working or studying in the field of application of brevibacteria in bioremediation processes.
• Data demonstrated here can be used by other researchers working or studying in the field of genome analysis.

## 1 Data

The draft genome sequence of *B. epidermidis* strain EZ-K02 constituted a total of 65 contigs (> 500 bp) with 3,885,924 bp, and a G + C content of 64.2% (Table 1). The RAST server predicted 3443 coding sequences where 1436 coding sequences (42%) were annotated as seed subsystem features and 2007 coding sequences (58%) annotated as outside of the seed subsystem. In total 2457 and 986 coding sequences were assigned as non-hypothetical and hypothetical, respectively. The genome was shown to encode at least 3 rRNAs and 47 tRNAs. The strain EZ-K02 possesses a substantial number of genes which are responsible for nitrate/nitrite ammonification (for example, in case with nitrate released during nitrocellulose denitration) as well as for metabolism of aromatic compounds, including genes involved in benzoate, *p*-hydroxybenzoate, acetophenone, catechol, gentisate and several other compounds biodegradation. ​Numerous genes responsible for resistance to toxic compounds, including arsenic, cobalt and cadmium, were additionally detected. Hence, *B. epidermidis* EZ-K02 may have high importance in the field of development of several effective environmental biotechnologies, such as environmental bioremediation and wastewater treatment.

**Table 1:** Comparison of the genomic feature of *Brevibacterium epidermidis* EZ-K02 strain with various *Brevibacterium* strains. The information regarding the reference genomes was received from the EzBioCloud database [11].

***Organism***	***DB accession number***	***Isolation source***	***Contigs***	***Genome size (bp)***	***G + C (%)***	***CDSs***	***r + tRNA genes***
*B. epidermidis* EZ-K02	GCA_002573745.1	Wastewater	65	3,885,924	64.2	3443	3+47
*B. album* DSM 18261	GCA_000426445.1	Saline alkali soil	15	4,094,970	70.9	3559	9+59
*B. casei* S18	GCA_000314575.1	Human skin	43	3,664,641	68.1	3233	6+46
*B. epidermidis* NBRC 14811	GCA_001570805.1	Human skin	25	3,703,261	64.3	3261	3+48
*B. linens* SMQ-1335	GCA_001729525.1	Cheese	1	4,209,935	62.6	3863	12+49
*B. ravenspurgense* CCUG 56047	GCA_001584615.1	Human specimens	14	2,297,397	62.4	2022	6+43
*B. senegalense* JC43	GCA_000285835.2	Human stool	17	3,427,329	69.4	3058	3+46

## 2 Experimental design, materials and methods

**Fig. 1:**
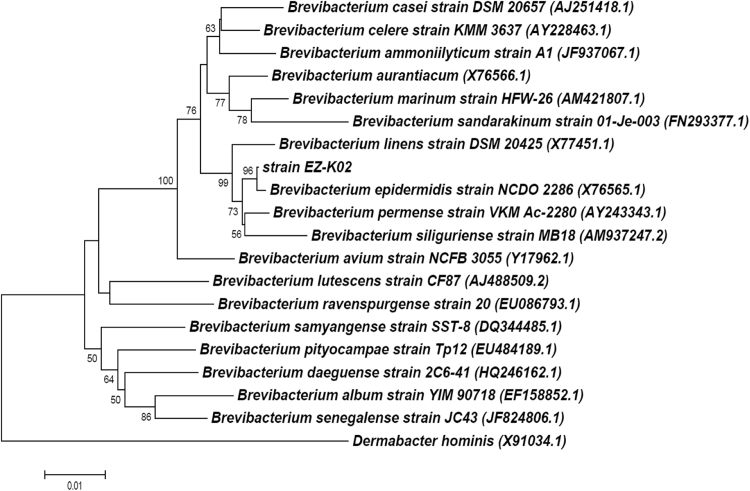
A phylogenetic tree based on 16S rRNA gene sequences demonstrating the relationship between *Brevibacterium epidermidis* EZ-K02 (NCBI accession number of 16S rRNA gene: MG050737) and the type strains from the LPSN site (www.bacterio.net). Analysis was conducted in MEGA7 [10] using the neighbor-joining method based on Jukes-Cantor evolutionary distances. The percentages of replicate trees in which the associated taxa clustered together in the bootstrap test (1000 replicates) are shown next to the branches. *Dermabacter hominis* was used as outgroup.

**Fig. 2:**
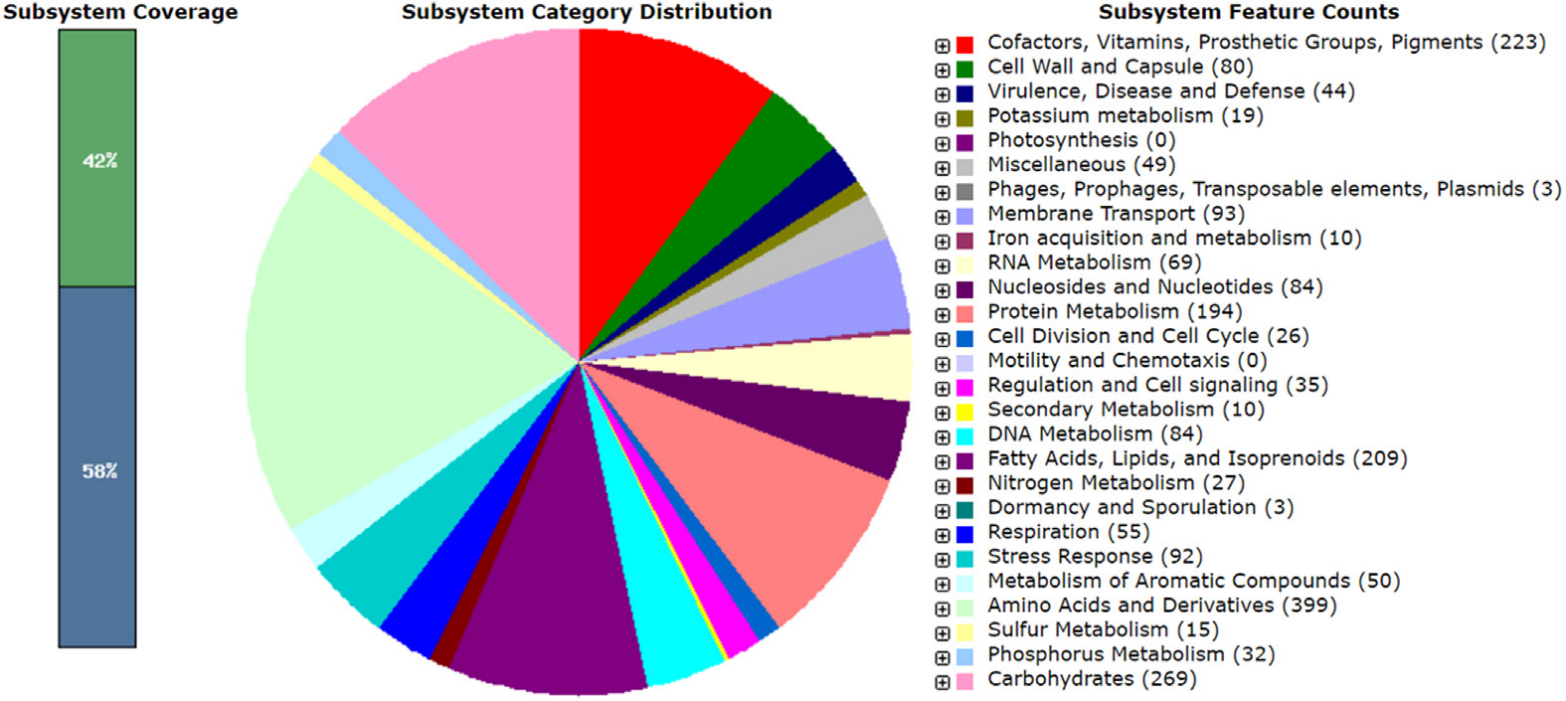
An overview of the subsystem categories assigned to the genome of *Brevibacterium epidermidis* EZ-K02. The whole genome sequence of the strain EZ-K02 was annotated using the Rapid Annotation System Technology (RAST) server [7]. The pie chart demonstrates the count of each subsystem feature and the subsystem coverage.

*B. epidermidis* EZ-K02 was isolated from nitrocellulose-contaminated wastewater environments, Kazan, the Republic of Tatarstan, Russia. Such industrial wastes are represented by large amounts of wastewaters contaminated with different dissolved chemical compounds and nitrocellulose powder particles. Also, these microbes are of high importance for the development of effective bioremediation strategies of various polluted environments [1], [2], [3]. The bacterium optimally grown on LB agar at +30 °C had been cultivated for 24–48 hours. Genomic DNA of the bacterial strain was then extracted and purified with a FastDNA spin kit (MP Biomedicals) and a FastPrep-24 homogenizer (MP Biomedicals) according to the manufacturer's protocol. Concentration and purity (A260/A280) of the extracted genomic DNA were measured with a NanoDrop 2000 spectrophotometer (Thermo Fisher Scientific) and stored at –20 °C until further processing. The bacterial strain EZ-K02 was morphologically identified and confirmed by PCR amplification using the primers UniBac27f, Bakt_805R and Univ1492r, followed by sequencing using an ABI PRISM 3130xl Genetic Analyzer (Applied Biosystems) and phylogenetic analysis (16S rRNA gene sequence;  1413 bp; Fig. 1). In order to perform whole genome analysis, DNA was fragmented using a Q800R2 Sonicator (Qsonica), and DNA library was then prepared with a NEBNext Ultra DNA Library Prep Kit for Illumina (New England Biolabs) according to the manufacturers’ protocols. Both efficiency of DNA fragmentation and DNA library preparation were monitored using a 2100 Bioanalyzer (Agilent) and a High Sensitivity DNA kit (Agilent). Further sequencing was performed with a high-throughput Illumina MiSeq platform (Illumina) at Joint KFU-Riken Laboratory, Kazan Federal University (Kazan, Russia) by a MiSeq Reagent Kit v2PE 500 cycles (Illumina). Briefly, sequence read quality was assessed using PRINSEQ lite version 0.20.4 [4], the genome was assembled using Velvet version 1.2.10 [5], and the ordering of contigs was achieved using Mauve version 2.4.0 [6]. The whole genome sequence of *B. epidermidis* was annotated using the Rapid Annotation System Technology (RAST) server [7]. The pie chart demonstrated the counts for each subsystem feature and the subsystem coverage (Fig. 2). The rRNA and tRNA genes were identified using RNAmmer 1.2 [8] and tRNA scan-SE 1.23 [9], respectively.
